# Biomedical Data Integration, Modeling, and Simulation in the Era of Big Data and Translational Medicine

**DOI:** 10.1155/2014/731546

**Published:** 2014-07-24

**Authors:** Bairong Shen, Andrew E. Teschendorff, Degui Zhi, Junfeng Xia

**Affiliations:** ^1^Center for Systems Biology, Soochow University, P.O. Box 206, Suzhou 215006, China; ^2^Cancer Institute, University College London, 72 Huntley Street, London WC1E 6BT, UK; ^3^Department of Biostatistics, University of Alabama at Birmingham, Birmingham, AL 35294, USA; ^4^Institute of Health Sciences, Anhui University, Hefei 230601, China

Advances in high throughput technologies, specially next-generation sequencing, have generated massive amounts of biological data. To take full advantage of these data and to extract as much information and knowledge from them as possible, we face many challenges. To help address and overcome these challenges and promote the application of informatics to translational research, we launched this special issue.

The biomedical data analyzed in this issue covers molecular, imaging, and clinical data. For instance, J. Shang et al. evaluated and compared multiple aligners for next-generation sequencing data, providing an important guide for biologists to select suitable aligners, and H. Li et al. proposed a method to identify mutated driver pathways in cancer. Y. Li et al. established a tissue type assignment method for glioblastoma multiforme by analyzing the magnetic resonance spectroscopy imaging data and tissue distribution information. F. Liu et al. applied multiple technologies to integrate the clinical and genomic information and to investigate their association for facilitating the diagnosis and treatment of colorectal cancer. X. Zhang et al. analyzed extensive clinical data, summarizing the disease spectrum, in China, and suggesting to pay more attention on disease prevention by promoting lifestyle changes.

In terms of translational research, two aspects, that is, biomarker discovery and drug design for diagnosis or treatment of diseases, were discussed based on computational studies. J. Huang and W. Yan identified micro-RNA biomarkers for sepsis and gastric cancer, based on miRNAs regulatory network analysis and systems biological approach, respectively. Y. Sun et al. successfully implemented big data technologies to a study of drug combinatorial effects. The Hadoop-based model showed higher efficiency and better performance than the traditional methods for the prediction of drug combination effects.

In this issue, we also compiled two technical works for biomedical data analysis. First is the work by H. Li et al., where they developed a hybrid support vector machine (SVM) model for privacy preserving data classification. The second is the work by J. Lei et al., where they made a systematic study on the usability of information technology, especially health information technologies, in China, by the analysis of publications during the past 30 years.

In addition to the analysis of static data, dynamic simulation is also important for biomedical data analysis. L. Jin performed a metadynamics simulation study of conformational transformation of HhaI methyltransferase and proposed that the induced fit model is necessary to understand the function of the studied molecule.

By launching this issue, we wish to give the readers a wider perspective on the future of data modeling and simulation and to leave the readers with the impression that informatics will be the key for successful translational research.

*Bairong  Shen* *Andrew  E.  Teschendorff* *Degui  Zhi* *Junfeng  Xia*

